# Adherence to Treatment and Factors Affecting Adherence of Epileptic Patients at Yirgalem General Hospital, Southern Ethiopia: A Prospective Cross-Sectional Study

**DOI:** 10.1371/journal.pone.0163040

**Published:** 2016-09-29

**Authors:** Temesgen Yohannes Hasiso, Tigestu Alemu Desse

**Affiliations:** 1 School of Pharmacy, Jimma University, Jimma, Ethiopia; 2 Clinical Pharmacy Department, School of Pharmacy, Jimma University, Jimma, Ethiopia; University of Rome Tor Vergata, ITALY

## Abstract

**Background:**

Non adherence of epileptic patients to antiepileptic medication often leads to an increased risk of seizures and worsening of disease, death and increased health care costs.

**Objective:**

to assess adherence to treatment and factors affecting adherence of epileptic patients at Yirgalem General Hospital, Southern Ethiopia.

**Methods and Materials:**

We conducted a cross-sectional study on epileptic patients from February 9 to 22, 2015. Data were collected from patients ≥18 years old. Adherence was measured using the eight-item Morisky’s medication adherence scale. All consecutive patients coming to epilepsy clinic during the study period were interviewed until the calculated sample size (210) was obtained. We collected patient demographics, perception about epilepsy and adherence to medication(s). We used chi-square tests and a binary logistic regression model for statistical analysis. Statistical significance was considered at P<0.05.

**Results:**

out of a total of 210 participants, 194 were willing to participate and were studied. Of the 194 participants, 109 (56.2%) were males. The mean age of the participants was 33.62±11.44 years; range 18 to 66 years. The majority, 123(63.41%), of the participants were taking two antiepileptic medications. Sixty two (32%) of the participants were adherent to their treatment. The most common reported reasons for non-adherence were forgetfulness 49(75.4%) and run out of pills 7(10.8%). Factors that affect medication adherence are epilepsy treatment for <1 year (P = 0.011), epilepsy treatment for 1–3 years (P = 0.002), epilepsy treatment for 3–5 years (P = 0.007), being married (P = 0.006), grade 9–12 education (P = 0.028), college or university education (P = 0.002) and absence of co-morbidity (P = 0.008).

**Conclusions:**

The rate of adherence observed in this study was low. The most common reason for non- adherence was forgetfulness. Therefore, the hospital should devise strategies to improve adherence of epileptic patients at the hospital.

## Background

Epilepsy is one of the most common and widespread neurological disorders. The global burden is estimated to be 1% [1]affecting over 65 million people[2]. It has profound physical, psychological and social impacts with a greater impact on a person’s quality of life than other chronic diseases[3]. Epileptic patients may also have lower quality of life due to enormous social stigmas [4].

Epilepsy is a major public health problem in low and middle-income countries (LMICs) imposing a large economic burden on the health care system[1]. World Health Organization in 2005 reported that 80% epileptic patients lived in developing countries[1,5]. Despite a high prevalence of epilepsy in LMICs, most people do not receive appropriate treatment. This is due to limited knowledge, poverty, cultural beliefs, stigma, poor health delivery infrastructure, and shortage of trained health care workers[6]. Many Africans believe epilepsy is contagious. As a result of this, they are unwilling to help or touch the person who has fallen during seizure. This kind of belief worsens the stigma[1,7].

Non-adherence to antiepileptic medications has been reported to be high. Studies showed a high prevalence of seizure (21–45%) in patients who did not adhere to their antiepileptic medications[8]. Patients who are non-adherent to their medication are frequently hospitalized with prolonged lengths of stay, have repeated emergency department visits, and miss school or work frequently because of the seizure effects or out of fear of seizure occurrence[9]. More than half of epileptic patients have poor seizure control due to non-adherence to medications. Studies showed a high rate of road accidents resulting in fracture, head injury and sudden unexpected death in patients with uncontrolled seizure attributed to non- adherence[10].

In developing countries, adherence of patients with chronic diseases is lower than 50%. The magnitude and impact of poor adherence in these countries is assumed to be even higher due to limited health resources and inequities in access to health care[11]. More than 30% of people with epilepsy do not attain full seizure control even with the best available treatment regimen. Failure to have a controlled seizure in such significant proportion of epileptic patients is attributed to poor adherence to medication(s)[12].

The patients’ beliefs about cause of epilepsy and preference to the treatment modality are important factors influencing epilepsy treatment. Patients’ own attitudes towards the treatment are also equally important in ensuring success of treatment and adherence[13]. Failure to adhere through forgetfulness, misunderstanding, or uncertainty about clinician’s recommendations, or intentionally due to their own expectations of treatment, side-effects, and lifestyle choice are found to be the reasons for non-adherence[14].

In general, medication adherence in epileptic patients is vital to have a good treatment outcome. Therefore, examining extent of adherence and identifying the underlying causes for non-adherence are necessary to improve the overall quality of life of patients. This study will assess the extent of non-adherence to antiepileptic medications and the factors that contribute to non-adherence among adult epileptic patients at Yirgalem General Hospital (YGH), Southern Ethiopia.

## Research Methods and Participants

We conducted a cross section study among adult epileptic patients at Yirgalem General Hospital (YGH), Southern Ethiopia from February 9 to 22, 2015. The hospital is located in Yirgalem town, Sidama Zone, Southern Nations Nationalities and Peoples Region, 312 km South of Addis Ababa. Epileptic patients get follow up from epilepsy clinic of the hospital every month.

The research was approved by Ethical Review Committee of Jimma University. Official letter of permission to collect data was obtained from the clinical director of the hospital.

Written informed consent was obtained from each participant prior to the interview. The participants had a right to withdraw from interview at any time. We assured the participants for confidentiality of their information and we maintained the privacy of the respondents.

The sample size was determined using a single population proportion formula [15] with a 95% confidence level as follows: n_i_ = (z_α/2_)^2^pq / d^2^ where ni = sample size; z = 95% confidence interval with ά = 5%; P = estimated prevalence, 50%; q = 1-p and d = margin of error (5%). Substituting all the values resulted in n_i_ = (1.96)^2^(0.5) (0.5) / (0.05)^2^ = 384.

Since the total number of epileptic patients was less than 10,000, the following correctional formula was used. As a result, the exact sample size (nf) was calculated as nf = ni×N/ni+N and substituting the variables resulted in nf = 384×379 /384+379 = 191. To compensate the non-response rate, 10% of the total sample size was taken and the final sample size obtained was 210.

Out of a total of 210 participants, 194 had complete data and were studied. The main outcome of this study was adherence to antiepileptic medication(s). All consecutive patients coming to epilepsy clinic for their regular follow up during the study period were interviewed until the desired sample size (210) was obtained. To maintain the quality of the data, data collectors were trained; the English version of the questionnaire was translated to local language and back translated to English. The data collection tool was also pretested.

Participants included in the study were epileptic patients ≥18 years old, epileptic patients who were on epilepsy treatment for at least three months, and patients who agreed and signed the informed consent form. We excluded patients with hearing problems and previously diagnosed psychiatric illness. Two trained pharmacists collected the data. We used a pretested structured questionnaire to collect data about sociodemographic characteristics, participants’ perception and belief about epilepsy. We used self-reported eight item Morisky medication Adherence Scale (MMAS-8) to collect data on adherence of the participants to their medications[16]. The scale (MMAS-8) consists of eight items with a score of “Yes” = 0 and “No” = 1 for the first seven items and a 5-point Likert response for the last item (eighth item). The items are summed and the scores are scaled as: 3-8-low adherence; 1-2-medium adherence and a score of zero as high adherence. In our study, patients with a score of low adherence and medium adherence were considered as non-adherent. We used data abstraction format to collect data on type of antiepileptic medication(s) patients were taking, duration of treatment, and co-morbid diseases.

### Statistical analysis

We analyzed the data using SPSS Version 16.0 (Chicago, SPSS Inc.). We used Chi- square tests to see the association between categorical variables and medication adherence. To examine factors affecting medication adherence, we made a multivariate logistic regression analysis. Variables with p-values <0.25 on a univariate logistic regression analysis were entered into a binary logistic regression model for multivariate analysis. Variables with p-value <0.05 were considered statistically significant.

### Operational definitions and definition of terms

#### Adherent

a patient was considered adherent to antiepileptic medication(s) if the MMAS-8 score was 0 and non-adherent if the score was 1 to 8.

## Results

### Sociodemographic characteristics of the participants

Out of a total of 210 participants, 194 were willing to participate and were studied. Sixteen patients refused to participate. Of the 194 participants, 109 (56.2%) were males. The mean age of the participants was 33.62±11.44 ranging from 18 to 66 years. Eighty two of the participants (42.3%) were in the age group between 18–29 years. The most common ethnic groups were Sidama, 68(35.1%), Wolayita, 28(14.4%) and Amhara, 27(13.9%). Protestantism 85(43.8%), Orthodox Christianity 51(26.3%) and Islam 37(19.1%) were the most common religions. Thirty two (16.5%) of the participants were married and 121 (62.4%) were single. The majority, 121(62.4%), of the participants were government employees.

More than half (55.2%) of the participants were urban residents. About 12% of the participants were illiterate. Seventy nine (40.7%) participants attended primary education and 48 (24.7%) participants attended college or university education (Table 1).

**Table 1:** Sociodemographic characteristics of epileptic patients at Yirgalem General Hospital.

Socio-demographic characteristics		Frequency	Percentage
Sex	Male	109	56.2%
	Female	85	43.8%
Age	<20	13	6.7%
	20–29	69	35.6%
	30–39	67	34.5%
	40–49	23	11.9%
	≥50	22	11.3%
Ethnicity	Sidama	68	35.1%
	Amhara	27	13.9%
	Wolayita	28	14.4%
	Gurage	18	9.3%
	Gedeo	13	6.7%
	Oromo	21	10.8%
	Others	19	9.8%
Religion	Orthodox	51	26.3%
	Muslims	37	19.1%
	Protestants	85	43.8%
	Catholic	18	9.3%
	Other	3	1.6%
	Illiterate	23	11.9%
Level of education	Primary school (grade 1–8)	79	40.7%
	Secondary school (grade 9–12)	44	22.7%
	College or university	48	24.7%
Marital status	Single	121	62.4%
	Divorced and widowed	41	21.1%
	Married	32	16.5%
Monthly Income	< 999 Birr	45	23.4
	1000–1999 Birr	84	43.3
	2000–2999 Birr	41	21.1
	>3000 Birr	21	12.4
Occupation	Daily laborer	17	8.8%
	Student	37	19.1%
	Government employee	121	62.37%
	Other	19	9.8%

The majority, 171(88.1%), of the participants had generalized tonic clonic seizures and the remaining had focal (partial) seizures. Of all the participants, 31(16.0%) had co-morbidity. Major depressive disorder was the most common co-morbidity; 20(64.5%). The remaining participants had hypertension (22.6%) and HIV (12.9%) infection.

### Perceptions of the participants towards their Disease

Sixty three participants (32.5%) perceived that their illness is due to a neurological disorder. Forty (20.6%) and 50(25.8%) participants perceived their illness as hereditary and evil spirit respectively. Forty one (21.1%) of the participants perceived that their illness is due to accident.

**Fig 1:**
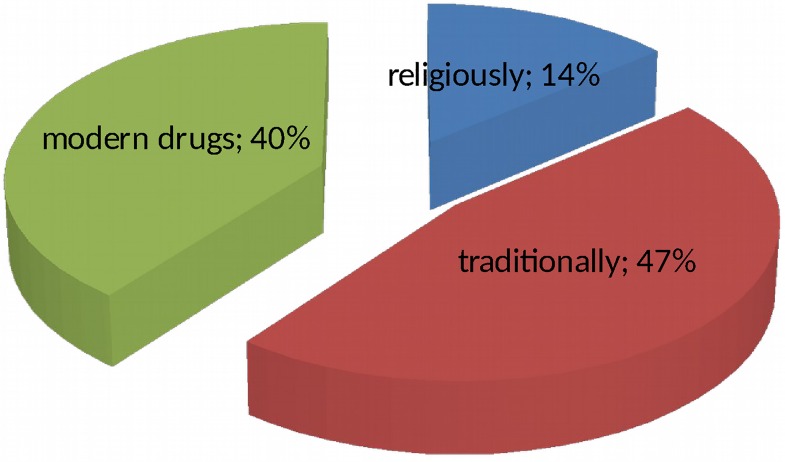
Perception of the patients about how the disease will be cured at Yirgalem General Hospital.

One hundred thirty four (69.1%) of the participants thought their illness (epilepsy) was serious and 43(22.8%) responded that their illness was a curable disease. The remaining 17(8.8%) of patients perceived that epilepsy is neither serious nor curable. Among respondents who responded epilepsy is curable, 20(46.5%), of them perceived it will be cured with traditional medicines, 17(39.5%) perceived it will be cured with modern medicines and 6(14.0%) perceived that it will be cured with religious interventions, Fig 1.

More than half, 121(62.3%), of the study participants reported that their epilepsy was not controlled after starting treatment. Ninety four (48.5%) of the participants reported that they had no improvement after starting treatment. Ninety two (47.4%) reported they had improvement after starting treatment. The remaining 8(4.1%) reported that they were not sure whether they had improvement or not.

### Treatments and adherence of patients to antiepileptic medication(s)

**Fig 2:**
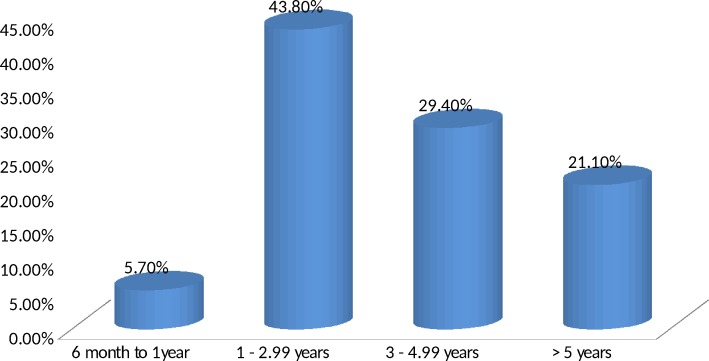
Duration of epilepsy treatment of epileptic patients at Yirgalem General Hospital.

About half, 98(50.5%), of the participants have been taking antiepileptic medication(s) for 3 years and above. Eleven (5.7%) participants have been taking antiepileptic medication(s) for <1year, Fig 2.

The majority of the participants, 123(63.4%), have been taking two antiepileptic medications. Phenobarbital and carbamazepine combination **(**57.7%) was the most common prescribed combination therapy. The remaining, 71(36.6%), of the participants were on monotherapy. Phenobarbital (19.6%) and phenytoin (10.8%) were the most common prescribed anti-epileptic medications as monotherapy (Table 2).

**Table 2:** Antiepileptic medications prescribed to treat epilepsy at Yirgalem General Hospital.

Antiepileptic medication	Frequency (%)	X2	P value
Phenobarbital	38(19.6)	5.520	0.238
Carbamazepine	12(6.2)		
Phenytoin	21(10.8)		
Phenobarbital + carbamazepine	112(57.7)		
Phenytoin + carbamazepine	11(5.7)		

According to MMAS-8 score, 70(36.1%), 62 (32.0%), and 62(32.0%) of the participants had a score of low adherence, medium adherence and high adherence respectively. Merging participants with low adherence and medium adherence together as non-adherent; 132(68.0%) epileptic patients were non-adherent to their antiepileptic medication(s). The remaining, 62(32.0%) of the participants were adherent to their antiepileptic medication(s).

**Fig 3:**
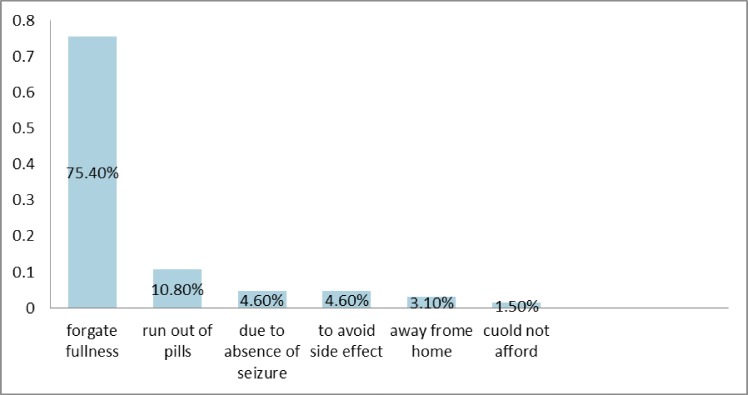
Self-reported reasons for medication non-adherence among epileptic patients at Yirgalem General Hospital.

Two third of the participants, 129(66.5%), took their medication as prescribed in the last one month. The most common reported reason for non-adherence was forgetfulness, 49(75.4%) as shown on Fig 3.

Prevalence of non-adherence was highest (100%) among patients ≥60 years old (Table 3). Divorced and widowed patients had higher rate of non-adherence as compared to married and single patients. On a chi-square analysis factors that have a statistically significant association with adherence are marital status (p < 0.001), level of education (P < 0,001), monthly income (p = 0.008), occupation (p < 0.001) and seizure control status (p < 0.001).

**Table 3:** The effect of socio-demographic characteristics on adherence of epileptic patients at Yirgalem General Hospital.

Variables		Adherence status		X2	P-value
		Adherent (%)	Non-adherent (%)		
Sex	Male	39 (35.8)	70 (64.22)	1.670	0.196
	Female	23 (27.1)	62 (72.94)		
Age	<20	3 (26.8)	10 (73.2)	18.41	0.002
	20–29	19 (9.8)	50 (25.8)		
	30–39	34 (44.1)	33 (55.9)		
	40–49	6 (26.1)	17 (73.9)		
	50–59	1 (7.7)	12 (92.3)		
	≥60	0	9 (100)		
Religion	Orthodox	19 (37.3)	32 (62.8)	0.963	0.915
	Muslim	11 (29.7)	26 (70.3)		
	Protestant	26 (30.6)	59 (69.4)		
	Catholic	5 (27.8)	13 (72.2)		
	Others	1 (33.3)	2 (66.7)		
Marital status	Married	19 (59.4)	13 (40.6)	20.451	< 0.001
	Single	39 (32.2)	82 (67.8)		
	Divorced	2 (8.0)	23 (92.0)		
	Widowed	2 (12.5)	14 (87.5)		
Residence	Rural	33 (37.2)	54 (62.8)	2.587	0.108
	Urban	29 (26.9)	78 (73.2)		
Level of education	Illiterate	2 (8.7)	21 (91.3)	32.164	< 0.001
	Primary school(1–8)	15 (17.7)	63 (82.3)		
	Secondary school(9–12)	15 (33.3)	30 (66.7)		
	College or university	30 (62.5)	18 (27.5)		
Occupation	Daily laborer	2 (11.8)	15 (88.2)	43.104	< 0.001

	Student	28 (75.7)	9 (24.3)		
	Government employee	30 (24.8)	91 (73.2)		
	Other	6 (31.6)	13 (68.4)		
Monthly income	< 999	12 (26.2)	33 (73.8)	11.764	0.008
	1000–1999	20 (18.5)	64 (71.5)		
	2000–2999	16 (47.8)	25 (52.2)		
	>3000	14 (58.3)	10 (41.7)		
Seizure control status	Controlled	60 (82.2)	13 (17.8)	157.577	<0.001
	Uncontrolled	6 (5.0)	115 (95.0)		

Patients living in urban area, patients that attended higher education and government employees had good adherence.

On a multivariate logistic regression model (Table 4), independent factors that affect medication adherence in epileptic patients are epilepsy treatment for <1 year (Adjusted Odds Ratio (AOR)) = 11.3, 95%CI: 1.8–72.8, P = 0.011), epilepsy treatment for 1–3 years (AOR = 6.1, 95%CI: 2–19.1, P = 0.002), epilepsy treatment for 3–5 years (AOR = 5.3, 95%CI: 1.6–17.6, P = 0.007), being married (AOR = 6.3,95%CI: 1.7–23.2, P = 0.006), grade 9–12 education (AOR = 6.3, 95%CI: 1.2–34.2, P = 0.028), college or university education (AOR = 13.3, 95%CI: 2.5–70.1, P = 0.002) and absence of co-morbidity (AOR = 6.3, 95%CI:1.6–24.7,P = 0.008).

**Table 4:** Independent factors affecting adherence of epileptic patients to antiepileptic medications at Yirgalem General Hospital.

Variable category		Adherent		P-Value	AOR(95%CI)
		Yes (n)	No (n)		
Duration of Epilepsy treatment in years	<1	5	4	0.011	11.3(1.8–72.8)
	1–3	33	54	0.002	6.1(1.9–19.1)
	3–5	23	34	0.007	5.3(1.6–17.6)
	>5	5	36		1
Marital status	Single	41	80	0.100	2.5(0.8–7.2)
	Married	19	13	0.006	6.3(1.7–23.2)
	Divorced and widowed	6	35		1
Educational Status	Illiterate	2	21		1
	Grade1-8	17	61	0.185	3(0.59–15.2)
	Grade 9–12	17	28	0.028	6.3(1.2–34.2)
	College or university education	30	18	0.002	13.3(2.5–70.1)
Co-morbidity	Yes	3	28		1
	No	66	100	0.008	6.3(1.6–24.7)

## Discussion

We found that the majority of the participants had uncontrolled epilepsy. Almost half (48.5%) of the participants reported that their epilepsy did not improve despite treatment. The majority of the participants were on phenobarbital and carbamazepine combination therapy. The majority of the participants were non adherent to their medication(s). The major reason for non-adherence was forgetfulness (75.4%). Independent predictors of antiepileptic medication(s) adherence are epilepsy treatment for <1 year, epilepsy treatment for 1–3 years, epilepsy treatment for 3–5 years, being married, grade 9–12 education, college or university education and absence of co-morbidity.

There is no single accepted ‘gold standard’ measure of medication adherence. Each method has its own drawbacks. There is no consensus standard on what constitutes adequate adherence in epileptic patients on antiepileptic medications[17].

In our study, adherence to antiepileptic medications was 32%. The adherence rate in our finding was lower than the findings from Brazil; 66.2%[18], Saudi Arabia; 61.7%[19], Palestine; 36% [20] and England; 41%[21]. In general, previous studies on medication adherence among epileptic patients reported adherence rates ranging from 36% to 70.8%[8,9,18–21].The reason for low rate of adherence in our set up may be due to poor patient awareness about epilepsy, the role of antiepileptic medication, and adherence to antiepileptic medication. In our finding, about 46.4% of the participants perceived that epilepsy is caused by evil spirit and hereditary inheritance which in turn might have affected their belief to modern drug therapy. In addition to what is afore stated, about 63.4% of the participants were taking 2 antiepileptic medications which may cause pill burden to patients and this may in turn affect adherence. As we are living in a third world country, patients have a very low monthly income to afford antiepileptic medications. This might have contributed to low level of adherence in our set up. Self-report may also affect the level of data obtained about medication adherence. In general, the differences in the levels of adherence between our finding and the findings from other countries may be due to differences in patients’ attitude towards antiepileptic medications as there may be differences in culture, belief, level of education, and physicians approach to epileptic patients or the degree of medical and parental supports.

In Ethiopia, Getachew et al [22] reported that medication adherence of epileptic patients at Jimma University Specialized Hospital was 63.2%. This higher level of adherence as compared to ours may be due to differences in the level of patient care and epilepsy management. At Jimma University Specialized Hospital, services to epileptic patients are provided by specialist physicians, medical residents and post graduate clinical pharmacy students. In our study area, general practitioners and health officers provide the service.

Age and medication adherence are significantly associated (p = 0.002). All patients (age ≥60 years) were non-adherent to their medications. This finding is similar to the reports from China (8). This may be due to the fact that elderly patients have more difficulty in following instructions owing to cognitive impairment or other physical difficulties such as problems in swallowing tablets, opening drug containers or handling small tablets. This is supported by the reports by Benner et al.; Jeste et al. and Cooper et al.[23–25].

Monotherapy is the gold standard epilepsy management. About 30% of patients become seizure-free with the administration of antiepileptic monotherapy[11]. A study in Saudi Arabia [19] showed that the number of antiepileptic medications used and level of medication adherence were significantly associated which is inconsistent with our finding.

We found that duration of epilepsy treatment and adherence were significantly associated.

This is in line with the findings by Kyngas [26] where the duration of the disease was significantly related to medication adherence. In contrast, Gabr et al [19] reported that duration of epilepsy and adherence were not significantly associated.

Married patients had a higher level of adherence as compared to divorced and widowed patients (p <0.006). Better adherence in married patients may be due to support of the partner in adhering to the prescribed medication(s) and instructions given by health care professionals. Chigier [27] also reported that the parent, other family member and friend support is considered as the corner stone to medication adherence among adolescents with epilepsy. Adolescents often want to talk with family members and friends about their personal issues [28] which may have a positive impact in improving adherence. Educational level above grade eight is significant predictor of good adherence. As educational level increases, patients’ awareness about the disease and the importance of adhering to medications will increase.

Kyngas [26] reported that discomfort from treatment, expense of treatment, decisions based on personal judgments about the effectiveness of the proposed treatment, maladaptive coping styles (e.g., denial of illness), or mental disorders have been considered the common reasons for non-adherence. We found that forgetfulness (75.4%) was the most common cause of non-adherence. This is supported by a number of studies. For example, Liu et al. [8]found that the primary reason for non-adherence was forgetfulness in 69.6% of his studied group of patients. Johnbull et al [29] reported that in more than 40% of the patients, the cause of non-adherence was forgetfulness. Paschal et al. [30]also reported that ‘‘forgetfulness” was the major reason for non-adherence. In our study, forgetfulness as the cause of non-adherence is higher than the finding from Brazil;47.5% [18] which may be explained by the difference in the level of care given to patients.

In our study duration of epilepsy treatment for <1 year, epilepsy treatment for 1–3 years, epilepsy treatment for 3–5 years, being married, grade 9–12 education, college or university education and absence of co-morbidity are independent factors that affect medication adherence. Our study has some limitations: all epileptic patients having follow up at the hospital had no equal chance of being included in the study as we took all consecutive patients that had follow up within two weeks period. We did not include epileptic patients that might have follow up by traditional healers and that might be absent from the hospital for regular follow up. The diagnosis of epilepsy was made by treating physicians. We collected data from patients diagnosed with epilepsy by physicians and the researchers did not reconfirm the diagnosis.

## Conclusions

We found that the rate of adherence in this study was low. The most common reasons for non- adherence were forgetfulness followed by run out of pills. The majority of the patients reported that their epilepsy was not controlled after starting treatment. Nearly two third of the patients were on two antiepileptic medications. Epilepsy treatment for <1 year, epilepsy treatment for 1–3 years, epilepsy treatment for 3–5 years, being married, educational level grade 9–12, college or university education and absence of co-morbidity were the factors that independently affected adherence. Therefore, we recommend zonal health department, the hospital, and health care providers to devise strategies to improve medication adherence and epilepsy control of patients at the hospital.
